# 1303. Making Infectious Disease Fellowship More Rashional: A Needs Assessment of Program Directors

**DOI:** 10.1093/ofid/ofac492.1134

**Published:** 2022-12-15

**Authors:** Danica Rockney, Sean Tackett, Michael T Melia

**Affiliations:** Johns Hopkins University, Baltimore, Maryland; Johns Hopkins Bayview Medical Center, Baltimore, Maryland; Johns Hopkins University, Baltimore, Maryland

## Abstract

**Background:**

Many infectious diseases present with rashes. Despite this, limited data have been published regarding dermatology training in infectious disease (ID) fellowships. We aimed to understand the perceived importance, current effectiveness, and existence of dermatology curricula for ID fellows in the United States (US).

**Methods:**

Over four weeks in March-April 2022, we emailed US ID fellowship program directors (PDs) individualized links to a Qualtrics survey. Up to two reminder emails were sent. Using NRMP Match Results and Data, we identified 151 PDs. Email addresses were found for 147 PDs. Descriptive data analysis was performed. This study was IRB exempt.

**Results:**

Response rate was 91/147 (62%). Most PDs (79/91, 87%) felt like it was very-to-extremely important for ID fellows to know basic dermatologic manifestations of infections (Image 1), though 91% (82/90) felt that they were only slightly-to-moderately effective at preparing fellows for this (Image 2). Most (55/90; 61%) programs do not have a formal dermatology curriculum for their fellows. Lack of faculty capacity, lack of reliable resources, and other topics taking priority were most commonly selected as significant barriers to implementing a dermatology curriculum (Image 3). Of the programs that do not have a dermatology curriculum, 76% (41/54) of PDs were interested in incorporating an externally produced dermatology curriculum with synchronous lectures (81%), asynchronous content (78%), and flipped classroom guides (72%) being the most favored educational strategies. For programs that currently have a dermatology curriculum (35/90, 39%), 67% (22/33) of PDs felt their fellows found their current dermatology curriculum very-to-extremely valuable.

**Figure:**
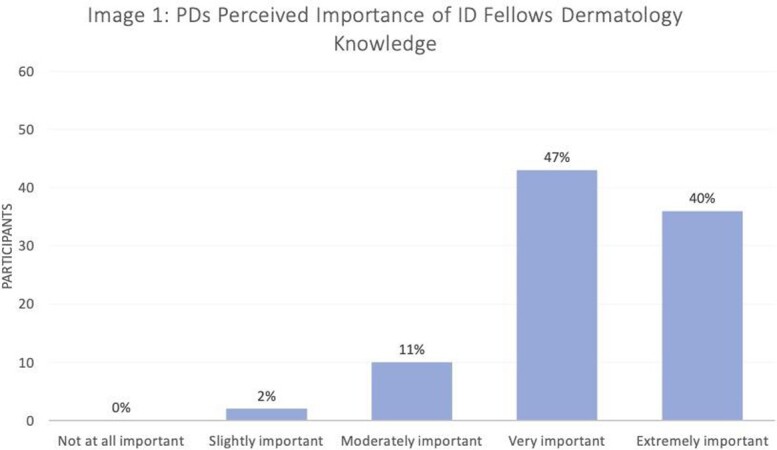

**Figure:**
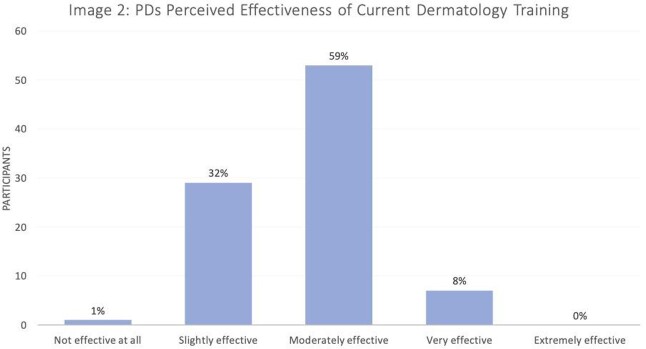

**Figure:**
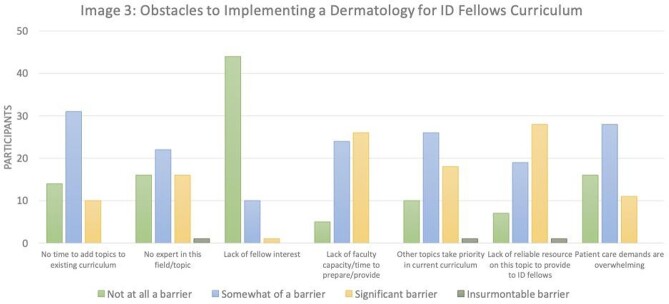

**Conclusion:**

These data indicate that current ID training often does not include formal dermatology-ID training. This data, along with a planned focus group of fellows, will help inform the development of a dermatology-ID curriculum to help address this content gap and improve ID fellowship education.

**Disclosures:**

**All Authors**: No reported disclosures.

